# *Arabidopsis thaliana* organelles mimic the T7 phage DNA replisome with specific interactions between Twinkle protein and DNA polymerases Pol1A and Pol1B

**DOI:** 10.1186/s12870-019-1854-3

**Published:** 2019-06-06

**Authors:** Stewart A. Morley, Antolín Peralta-Castro, Luis G. Brieba, Justin Miller, Kai Li Ong, Perry G. Ridge, Amanda Oliphant, Stephen Aldous, Brent L. Nielsen

**Affiliations:** 1 Department of Microbiology & Molecular Biology, Brigham Young University, 3130 Life Sciences Building, 4007 LSB, Provo, UT 84604 USA; 2 Langebio-Cinvestav Sede Irapuato, Km. 9.6 Libramiento Norte Carretera. Irapuato-León, 36821 Irapuato, Guanajuato Mexico; 3 Department of Biology, Brigham Young University, 4007 LSB, Provo, UT 84604 USA

**Keywords:** Arabidopsis, Organellar DNA replication, Yeast-two-hybrid, Direct-coupling-analysis, Thermophoresis

## Abstract

**Background:**

Plant chloroplasts and mitochondria utilize nuclear encoded proteins to replicate their DNA. These proteins are purposely built for replication in the organelle environment and are distinct from those involved in replication of the nuclear genome. These organelle-localized proteins have ancestral roots in bacterial and bacteriophage genes, supporting the endosymbiotic theory of their origin. We examined the interactions between three of these proteins from *Arabidopsis thaliana*: a DNA helicase-primase similar to bacteriophage T7 gp4 protein and animal mitochondrial Twinkle, and two DNA polymerases, Pol1A and Pol1B. We used a three-pronged approach to analyze the interactions, including Yeast-two-hybrid analysis, Direct Coupling Analysis (DCA), and thermophoresis.

**Results:**

Yeast-two-hybrid analysis reveals residues 120–295 of Twinkle as the minimal region that can still interact with Pol1A or Pol1B. This region is a part of the primase domain of the protein and slightly overlaps the zinc-finger and RNA polymerase subdomains located within. Additionally, we observed that *Arabidopsis* Twinkle interacts much more strongly with Pol1A versus Pol1B. Thermophoresis also confirms that the primase domain of Twinkle has higher binding affinity than any other region of the protein. Direct-Coupling-Analysis identified specific residues in Twinkle and the DNA polymerases critical to positive interaction between the two proteins.

**Conclusions:**

The interaction of Twinkle with Pol1A or Pol1B mimics the minimal DNA replisomes of T7 phage and those present in mammalian mitochondria. However, while T7 and mammals absolutely require their homolog of Twinkle DNA helicase-primase, *Arabidopsis* Twinkle mutants are seemingly unaffected by this loss. This implies that while *Arabidopsis* mitochondria mimic minimal replisomes from T7 and mammalian mitochondria, there is an extra level of redundancy specific to loss of Twinkle function.

**Electronic supplementary material:**

The online version of this article (10.1186/s12870-019-1854-3) contains supplementary material, which is available to authorized users.

## Background

Mitochondria and chloroplasts possess their own unique genomes. The endosymbiotic theory provides the leading explanation for the existence of mitochondria and chloroplasts in eukaryotic cells. In this theory, modern mitochondria and chloroplasts are the result of a symbiosis between ancient free-living α-proteobacteria and cyanobacteria with a host cell [1]. Over time, the endosymbiotic α-proteobacteria and cyanobacteria lost more and more of their DNA coding capacity to the nucleus of the host organism until they lost the ability to be free living. However, time has not removed all DNA from these organelles, leaving behind the mitochondrial and chloroplast genomes we are familiar with today. This DNA is not an artifact, it is fully functional with genes that are replicated, transcribed and translated to produce essential proteins for organelle function [2, 3]. The mechanisms in place for maintenance of this DNA are similar to bacteriophage systems and are much simpler than the mechanisms involved in eukaryotic nuclear DNA replication and bacterial chromosomal replication [4, 5].

Many plant organellar DNA replication proteins have been identified and characterized in various species [4, 6]. In this report we focus on the interactions between the Arabidopsis organelle DNA replication proteins Twinkle and DNA Polymerases 1A and 1B. We propose that a minimal functioning DNA replisome in plant chloroplasts and mitochondria consists of the DNA primase-helicase protein Twinkle along with DNA polymerase 1A or 1B. We also propose that SSB1 participates in this minimal replisome. This system has been described as the minimal mitochondrial DNA replisome in mammals [7], and mimics the replication machinery of T7 bacteriophage [8].

*Arabidopsis* Twinkle protein gets its name from the human homolog [9], which is similar to the bacteriophage T7 gp4 DNA primase/helicase protein. In humans this protein lacks DNA primase activity due to amino acid sequence differences in the primase domain [10]. In plants, this protein has both DNA helicase and primase activities [11, 12]. T7 gp4 protein functions as both a DNA helicase and primase and is central to the replication machinery of its genome [8]. Structurally, *Arabidopsis* Twinkle and T7 gp4 are very similar; however, *Arabidopsis* Twinkle has a slight extension between the primase and helicase domains when compared to phage gp4 as well as a longer N-terminal region.

Zinc fingers are typically associated with DNA binding domains, with many examples found in transcription factors. Zinc fingers may also be involved in protein folding and assembly, RNA packaging, and lipid binding [13]. *Arabidopsis* Twinkle protein, similar to the T7 gp4 protein, has a functional zinc finger domain. The zinc finger of T7 gp4 is required for functional primase activity [14]. In humans and mammals, amino acid changes including loss of cysteine residues in ancestral zinc finger motifs lead to a non-functional primase domain [15].

The ancestral origin of plant organellar DNA polymerases is convoluted. Initially, *Arabidopsis* proteins Polymerase 1A and 1B were designated Polymerase gamma 2 and 1, respectively, in reference to human mitochondrial DNA POLγ [16]. However, further studies disputed this characterization and found that the plant proteins have more in common with bacterial DNA polymerase I; hence the names Pol1A and Pol1B [17]. Furthermore, other studies frequently refer to these proteins as plant organellar DNA polymerases, or POPs [18]. Thus, three different names exist for the same protein. In this paper we refer to the proteins as DNA polymerase 1A and 1B. These are the only DNA polymerases known to function within the chloroplasts and mitochondria in plants. The phage T7 homolog of Pol1A and Pol1B is the gp5 protein. Although Pol1A and Pol1B share approximately 70% amino acid identity with each other, studies have identified several different characteristics for each. Parent et al. reported that Pol1B serves more of a role in DNA repair than Pol1A [17], and Pol1A replicates with more fidelity and is able to displace DNA more effectively than Pol1B [19, 20]. These previous works show that Pol1A and Pol1B are not merely redundant DNA polymerases but each has specific functions.

**Fig. 1:**
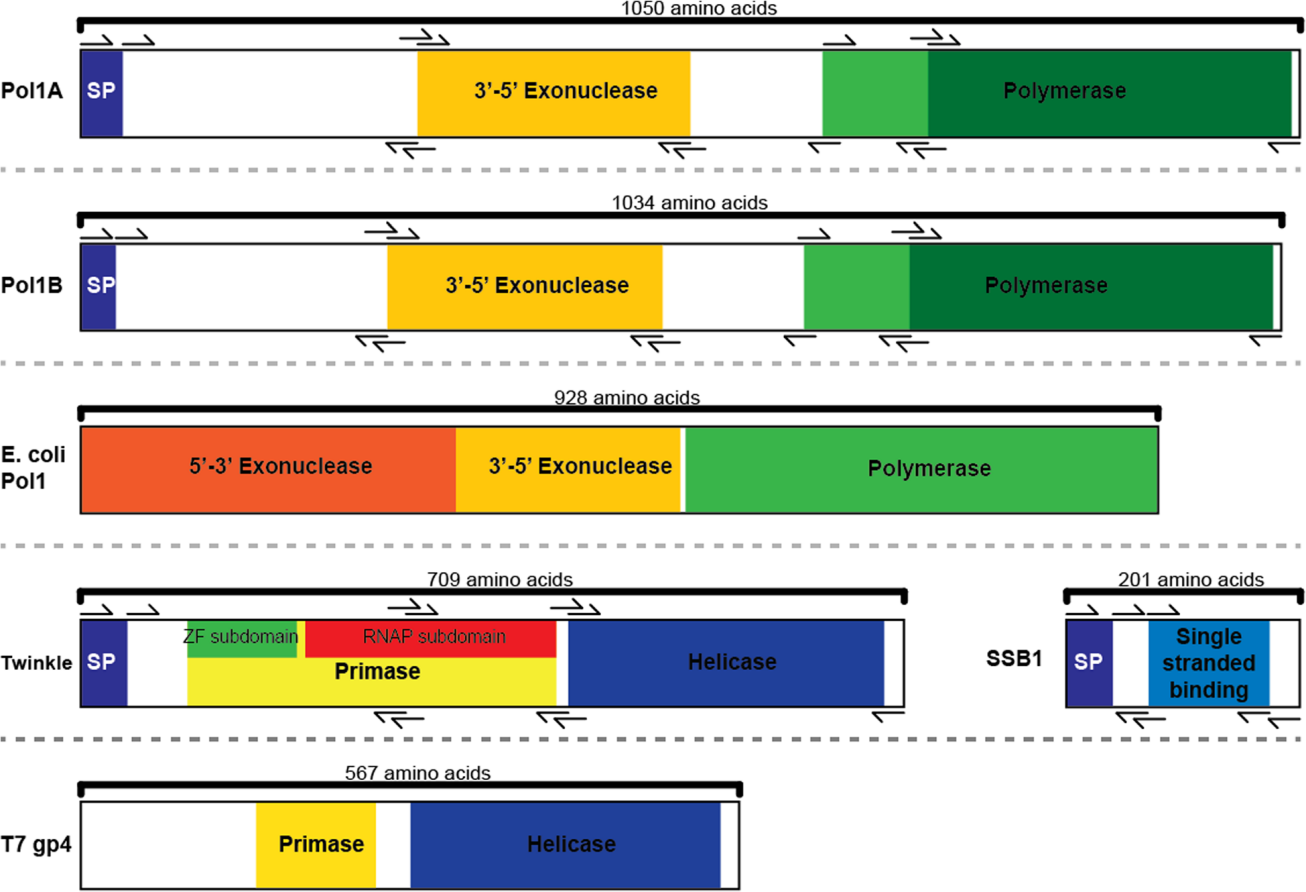
*Arabidopsis* Pol1A, Pol1B, Twinkle, SSB1, *E. coli* DNA polymerase I, and T7 gp4. “SP” stands for “signal peptide” as predicted by the SignalP server. Functional domains are illustrated based on results from the NCBI conserved domain database, Uniprot [57] databases and published works by Bernstein [58], Gray [15] and Richardson [59]. Signal peptide regions were predicted using the SignalP [60] server. With the exception of SSB1, these proteins are unusually large, especially when compared to the length of orthologous plant and animal proteins that function in the nucleus [61]. The light green region in Pol1A and Pol1B represents the border of the plastid-like DNA pol a domain which overlaps with the dark green region representing the more general DNA pol a domain. Genes are shown to scale in terms of cDNA and amino acid length. Black arrows represent PCR primers used to create truncations for each protein. Note that in our yeast-two-hybrid experiment, all potential truncations were tested against each other simultaneously by creating truncation libraries in haploid yeast which were mated and grown on selective media

There are a few structural differences between *E. coli* DNA polymerase I and *Arabidopsis* Pol1A and Pol1B. The most obvious is that Pol1A and Pol1B lack the 5′-3′ exonuclease domain found in *E. coli* DNA polymerase I. In its place is a long sequence of amino acids with no functional assignment. In *E. coli,* this 5′-3′ exonuclease activity is involved in removal of Okazaki fragments. Lack of this domain in the plant DNA polymerases suggests that primer removal has been replaced by another mechanism or by other proteins. Therefore, it is possible that as the plant DNA polymerases evolved, the 5′-3′ exonuclease domain was not maintained and became non-functional as mutations accumulated. The second difference between the plant and bacterial DNA polymerases is a stretch of amino acids that has been inserted between the 3′-5′ exonuclease domain and the polymerase domain (Fig. 1). In structural predictions this takes on a large looping structure.

SSB1 is a single stranded DNA binding protein with homologs in many other organisms. When making comparisons, this paper will refer to the T7 phage SSB1 homolog, gp2.5. An earlier study has shown that in humans, minimal mitochondrial DNA replisomes can be formed in vitro with just TWINKLE and POLγ [7]. Addition of mtSSB1 protein enhances this replisome. The interactions between these human proteins mimics the simple system found in T7 phage which consists of gp4 DNA primase-helicase, gp5 (DNA polymerase), and gp2.5 (SSB) proteins [8].

It stands to reason that plants utilize similar replisomes to maintain their organellar DNA. We propose that the minimal DNA replisome in plant organelles consists of similar components found in humans and utilized by T7 phage, namely Twinkle, Pol1A or Pol1B, and SSB1 proteins. In this paper, we will demonstrate the interactions of these proteins, focusing on the specifics of Twinkle and the DNA polymerases. We have identified one specific region of Twinkle with a predicted disordered structure, and we show that this region is key for Twinkle’s interactions with Pol1A and Pol1B.

## Results

We hypothesized that Pol1A, Pol1B, and Twinkle interact with each other. This was based on previous studies that detailed DNA replication machinery in T7 phage as well as in vitro studies of the human homologs of Twinkle, Po1A, Pol1B, and SSB1 [7, 8]. To test our hypothesis we used Direct Coupling Analysis (DCA), yeast-two-hybrid, and thermophoresis.

### Direct coupling analysis of Twinkle, Pol1A and Pol1B

**Fig. 2:**
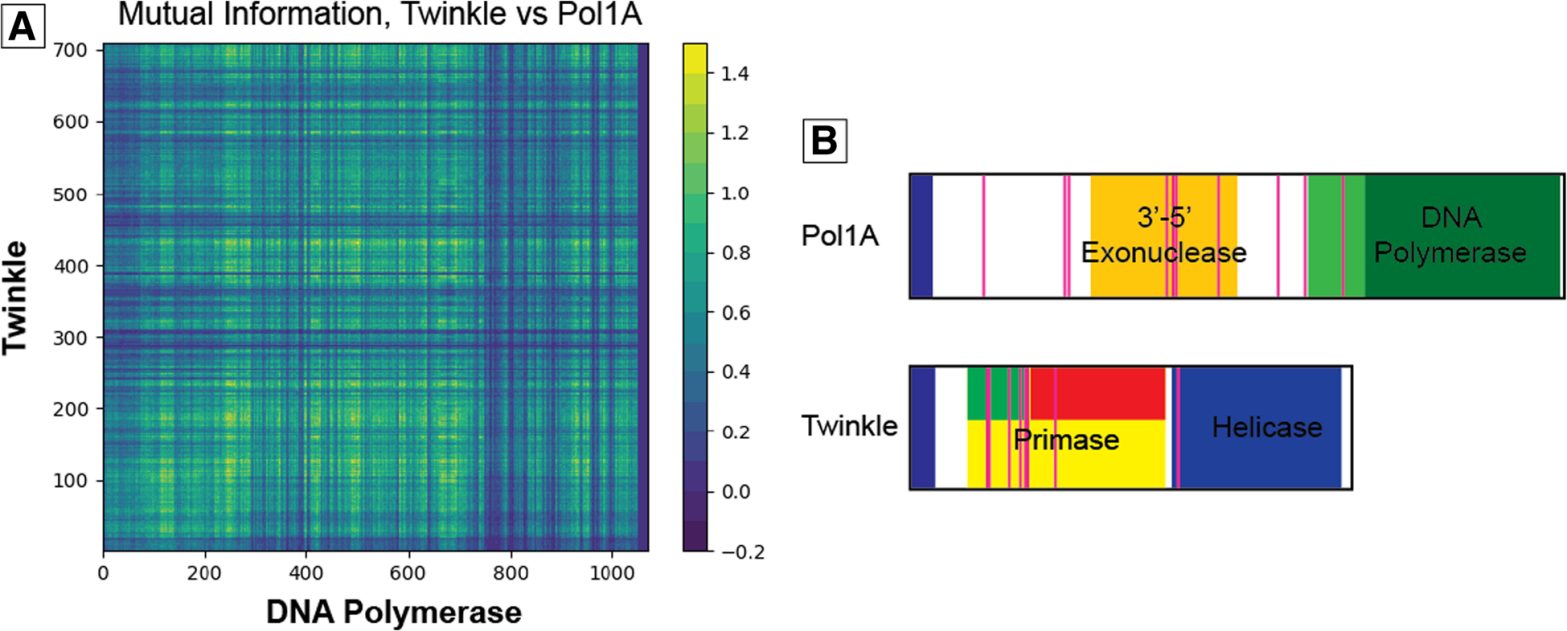
Heat map of residues most likely to interact based on Direct Coupling Analysis. (**a**) Heat map showing the likelihood of residues from Twinkle (y-axis) to interact with residues from Pol1A (x-axis) based on mutual information generated from DCA. This heatmap was generated using multiple sequence alignment data of Twinkle, Pol1A and Pol1B homologs. The final heatmap can be drawn using either Pol1A or Pol1B on the x-axis with near identical results. This heatmap displays Pol1A on the x-axis. The raw data used to make this heat map was analyzed to identify 10 residues in both the polymerases and Twinkle with the highest likelihood for interaction. (**b**) Location of the 10 residues identified from DCA analysis mapped to Pol1A, Pol1B, and Twinkle (vertical red lines). These residues were picked for point mutation analysis to see how they would affect the Polymerase-Twinkle interaction

Direct coupling analysis (DCA) is a bioinformatic technique that quantifies interactions between two positions in a sequence and can be used to evaluate evolutionary constraints by examining orthologous gene conservation [21]. In our case, we calculated the direct coupling of Twinkle and the DNA polymerase orthologs taken from 90 plant species. Although DCA is typically conducted on hundreds of protein sequences, since our model was intended to assess direct coupling in *Arabidopsis thaliana*, we limited the multiple sequence alignment to include only protein sequences originating in plant genomes. Furthermore, DCA is shown to systematically improve when sequence similarity between the protein sequences is less than 80% [21]. To ensure a balance between the number of sequences and potential biases incurred by sampling only a few species with very similar protein sequences, we included only a single protein sequence from each species, sampling from 90 available genomes from different species. From this data we generated a heat map and selected 10 residues from each protein with the highest likelihood of interaction. Of the 10 residues identified in Twinkle, 8 reside in close proximity to each other within the primase domain of Twinkle (Fig. 2). For the DNA polymerases, the top 10 residues are spread out much further throughout the protein. The exact position of each candidate residue is reported in Table 1.

**Table 1:** Top 10 residues likely to display interaction between Twinkle protein and Pol1A or Pol1B as predicted by Direct Coupling Analysis (DCA). Amino acid designations and tri-alanine mutants for each of these residues is also displayed

Twinkle		
Residue number	Amino acid	Tri-Alanine Mutant created
124	Glu	∆ 123,124,125
126	Cys	∆ 126,127,128
127	Pro	
159	Leu	∆ 158,159,160
177	Ala	∆ 176,177,178
185	Asp	∆ 184,185,186
189	Lys	∆ 188,189,190
233	Gly	∆ 232,233,234
430	Thr	∆ 429,430,431
431	His	
Pol1A/B		
Residue number	Amino acid (Pol1A/Pol1B)	Tri-Alanine Mutant created*
118	Val/Glu	∆ 117,118,119
248	Arg/Asp	∆ 247,248,249
255	Val/Glu	∆ 254,255,256
412	Pro/Lys	∆ 411,412,413
422	Glu/Asp	∆ 421,422,423
427	Leu/Lys	∆ 426,427,428
495	His/Phe	∆ 494,495,496
591	Val/Lys	∆ 590,591,592
634	Asn/Val	∆ 633,634,635
695	Thr/Gly	∆ 694,695,696

*Mutants were created for Pol1A and not Pol1B

### Yeast-two-hybrid analysis

**Fig. 3:**
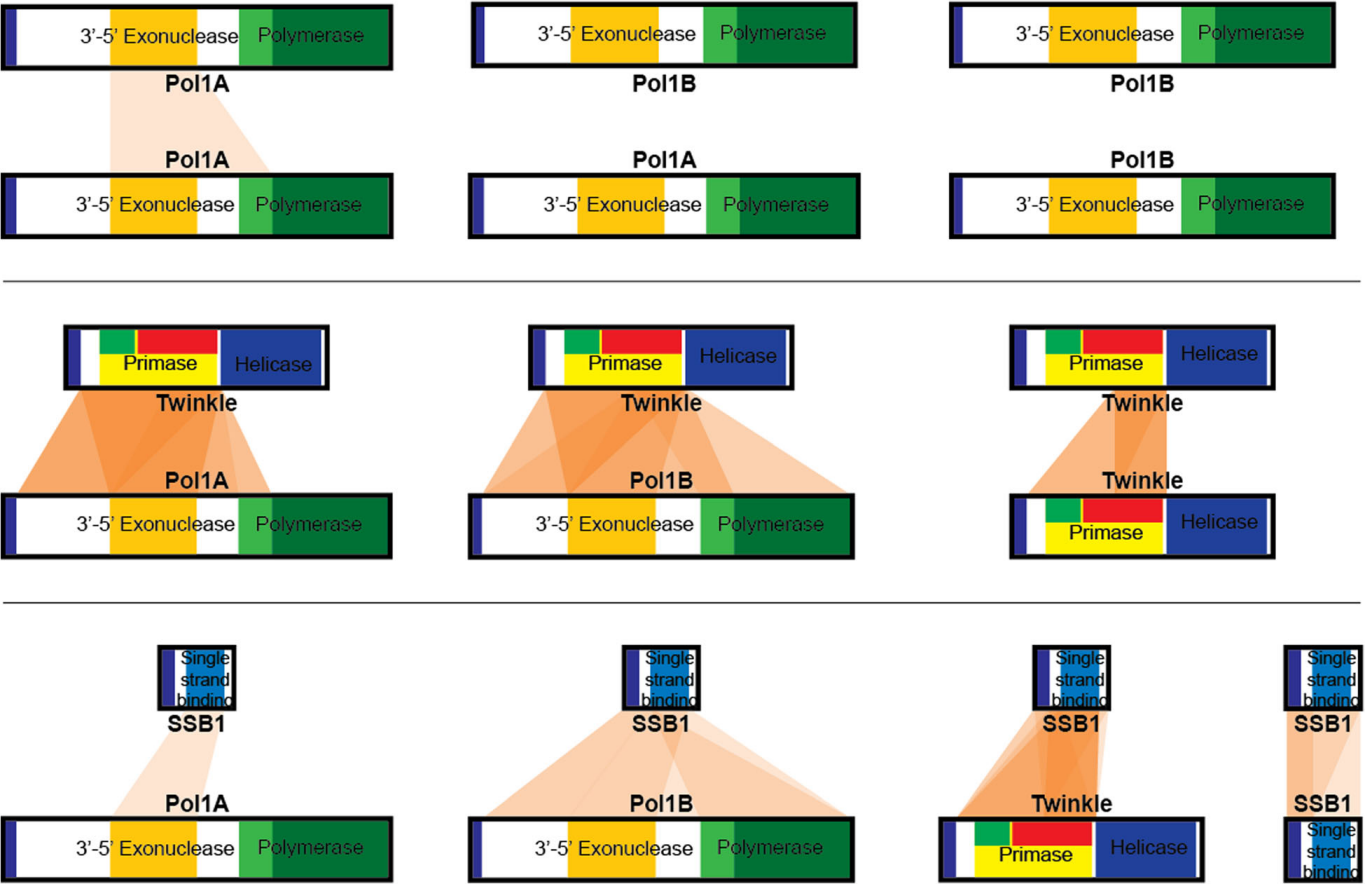
Summary of interactions after yeast library mating. Orange bridges between each protein represent unique interactions observed between specific gene truncations. Thus, higher numbers of unique interactions leads to darker bridges between proteins

To ensure that we did not miss any interactions, we created a series of protein truncations. The design of these truncations is shown in Fig. 1. All possible truncations were compiled into haploid yeast cells to create truncation libraries for Pol1A, Pol1B, Twinkle, and SSB1. Haploid yeast truncation libraries were mated and then plated on media selective for positive interactions. This allowed us to test every possible truncation of one protein against every possible truncation of another protein. The resulting interactions between the truncated protein constructs of Pol1A, Pol1B, Twinkle, and SSB1 are summarized in Fig. 3.

**Fig. 4:**
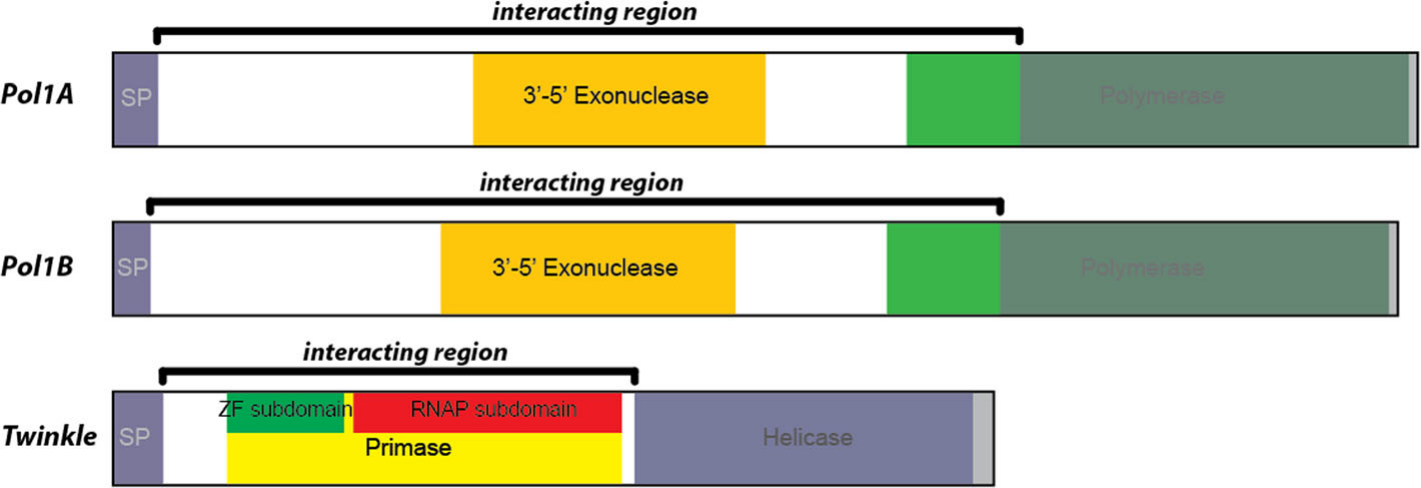
Regions of Pol1A, Pol1B, and Twinkle involved in the highest number of yeast-two-hybrid interactions. The regions highlighted above are still rather large and were selected for further truncation to specify exactly which regions of the protein led to positive interaction

Our library screen showed consistent interactions between specific regions of Twinkle and three other proteins; Pol1A, Pol1B, and SSB1. In particular, the region of Twinkle downstream from the predicted signal peptide and before the helicase domain appeared repeatedly in many interactions. A similar pattern occurs with the DNA polymerases; the region downstream from the predicted signal peptide and slightly inside the polymerase domain appear in many interactions. The interacting regions are highlighted in Fig. 4. These results support our hypothesis that the N-terminal region of Twinkle is responsible for interacting with the DNA polymerases. However, this does not necessarily indicate that only the N-terminal regions of the polymerases interact with Twinkle. The observed interacting regions of the polymerases are very large and eclipse several domains of the protein, not just the N-terminal region. Because the regions we identified in both proteins are quite large, we decided to further investigate the interaction between Twinkle and the polymerases by truncating these regions further.

**Fig. 5:**
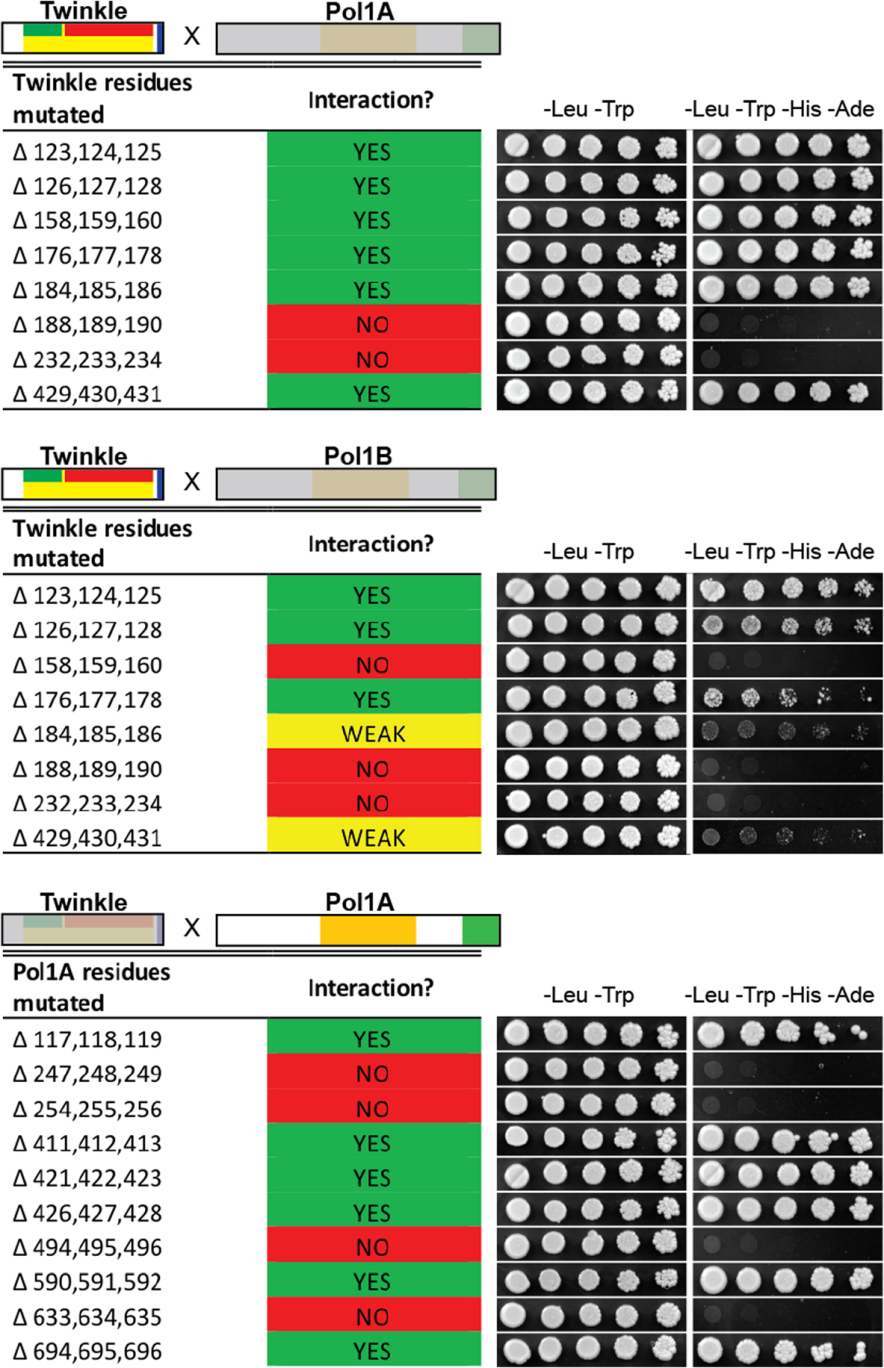
Results of tri-alanine substitution mutation analysis. Tri-alanine mutants were created in Twinkle and Pol1A to test for interaction disruption. Mutated residues were selected based off of DCA analysis results. While only two mutations caused a disruption of Twinkle with Pol1A, most mutations weakened or completely disrupted interaction with Pol1B. Mutations in Pol1A were much more distinct, either completely disrupting interaction with Twinkle, or failing to affect this interaction at all

We also mutated each of the 10 residues identified by DCA analysis and their immediate neighbors in Twinkle and Pol1A to determine whether they would disrupt interactions (Fig. 5). We chose to mutate Pol1A as our previous work showed more positive associations with Twinkle and therefore a loss of interaction would be more distinct. All mutations were made by substituting the original residues and their neighbors with alanine creating tri-alanine mutants. Because DCA analysis is based on gene alignments from many species, we mutated the candidate residues and their immediate neighbors to correct for imperfect positioning of critical interacting residues in Twinkle or Pol1A. Some of the candidate residues in Twinkle were so close together we were able to mutate both of them with one clone. Similar to the previous analysis, we observed much stronger interactions between Twinkle and Pol1A vs Pol1B (Fig. 5). We also detected more interaction disruptions between Pol1B and Twinkle due to point mutations. Based on the results of DCA and our initial yeast-two-hybrid analysis, we hypothesized that a relatively small region of Twinkle interacted with Pol1A and Pol1B.

### Truncating Twinkle

**Fig. 6:**
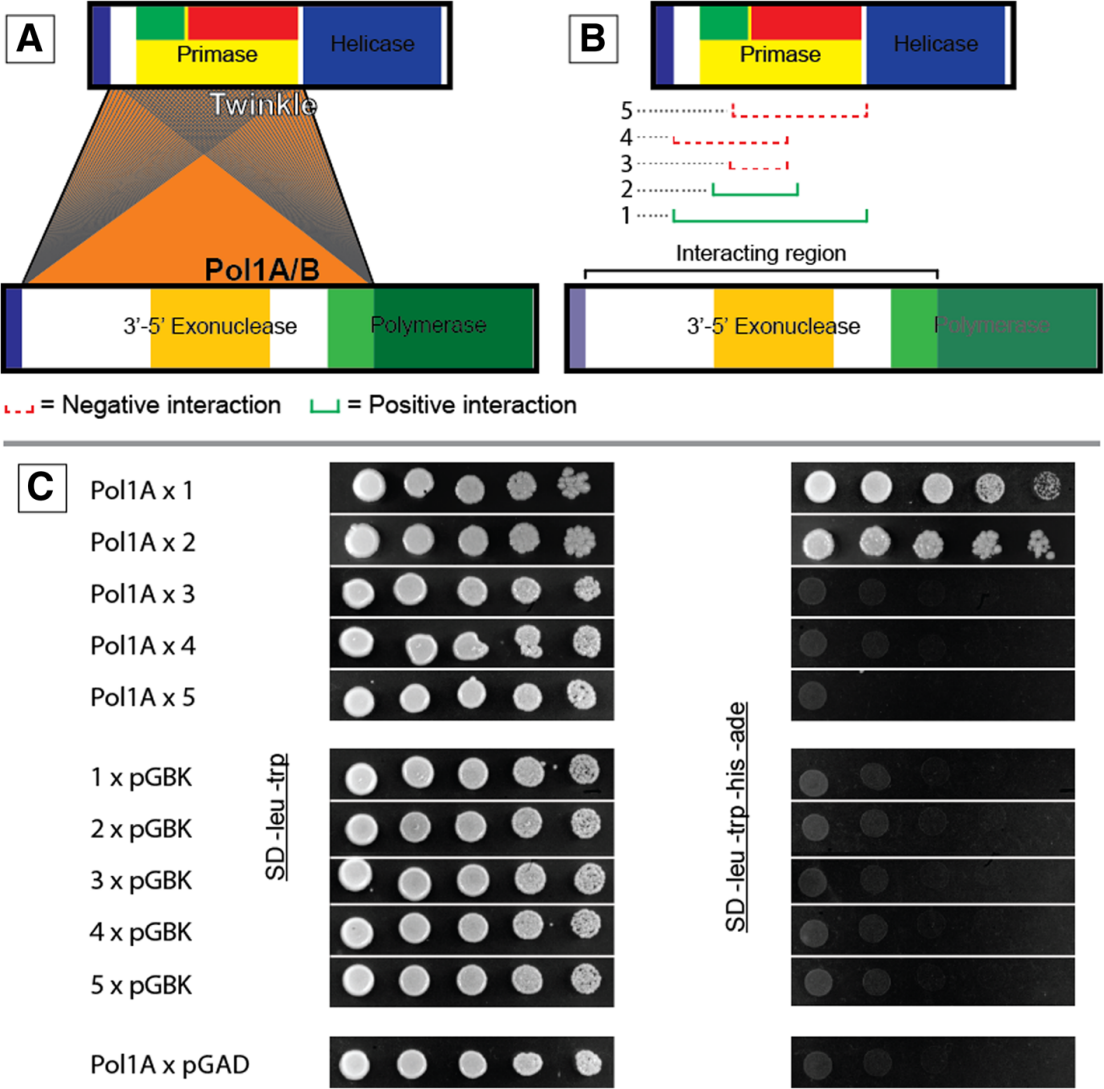
Design and results of Twinkle protein truncations. (**a**) Schematic showing the identified regions of Twinkle and Pol1A/B with a high number of positive interactions. Each line represents a 10 amino acid truncation from either the N-terminal or C-terminal side of Twinkle. (**b**) The smallest truncation of Twinkle that still interacted with Pol1A/B

To make Twinkle truncations we designed primers that shortened the protein in 10 amino acid increments from either the N-terminal or C-terminal side of the interacting region. We amplified and tested different truncations until the interaction was lost. After finding the maximum truncation from both the N-terminal and C-terminal regions that retained a positive interaction, we created a final truncation that combined these borders. Residues 120–295 of Twinkle make up the smallest truncation that maintains interaction with Pol1A or Pol1B, as illustrated in Fig. 6 for Pol1A (Pol1B results displayed in Additional file 4). This region does not reside in any predicted functional domain and further strengthens our hypothesis that the N-terminal region of Twinkle coordinates interaction with DNA polymerase.

### Truncating Pol1A and Pol1B

**Fig. 7:**
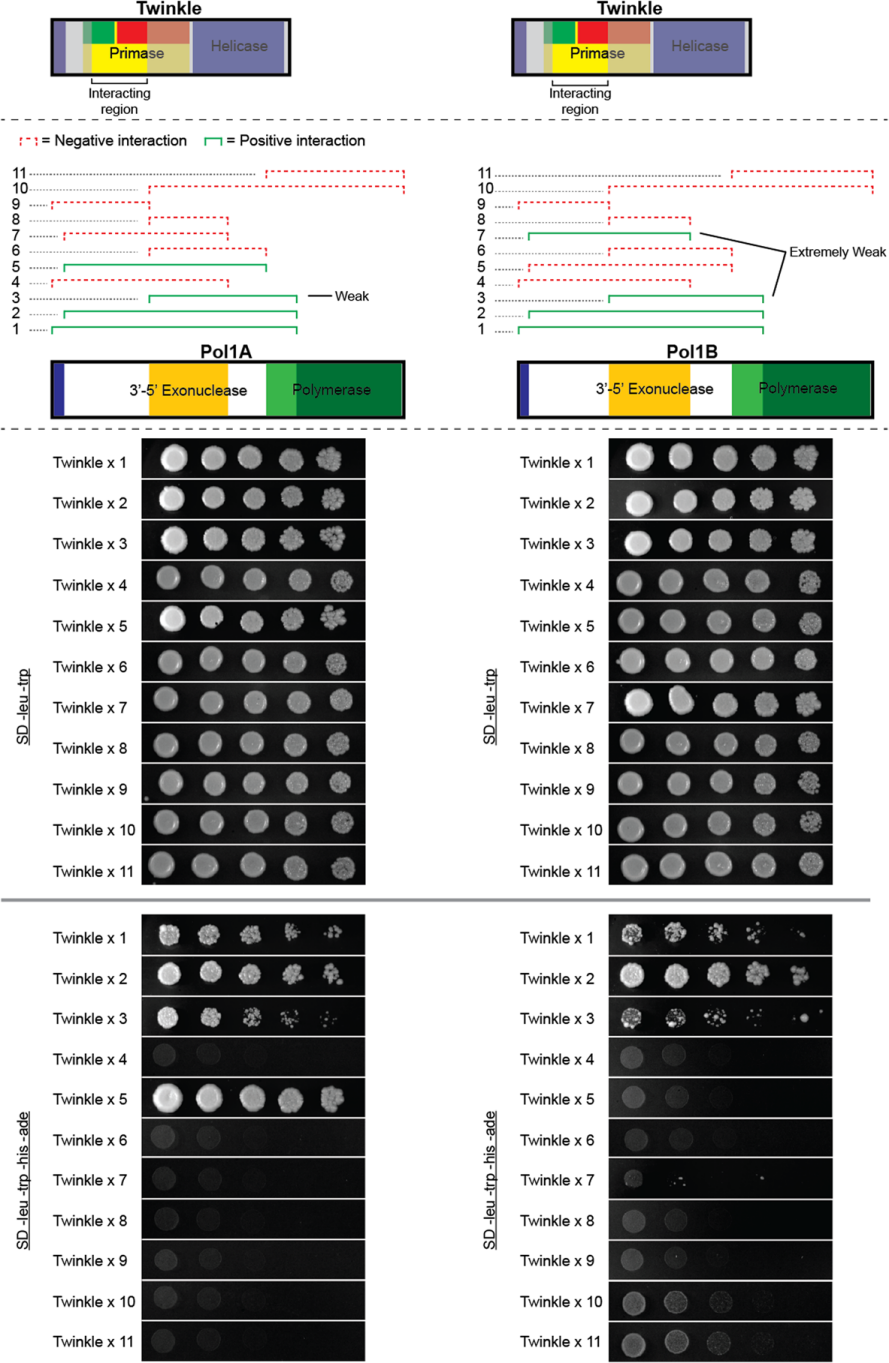
Truncations of Pol1A and Pol1B interacting with residues 120–295 of Twinkle. Truncations are numbered 1–12 and span Pol1A and Pol1B as designated above. Each truncation was tested for association with the interacting region of Twinkle and plated in 1:5 spot dilutions on selective and non-selective media as a control. Non-selective media is SD -leu -trp and the selective media is SD -leu -trp -his -ade. Note the stronger interaction between Pol1A and Twinkle versus Pol1B and Twinkle

We next truncated the DNA polymerase genes as we did with Twinkle in order to hone in on a specific interacting region. To do this we used the minimal region identified from the Twinkle truncations as a binding partner. Before designing primers for the 10 amino acid deletions, we produced truncations using the primers originally designed in our library approach (Fig. 1). The purpose was to identify a significantly smaller region of the DNA polymerases that would still interact with Twinkle in order to reduce the amount of effort required to produce dozens of 10 amino acid truncations. However, we found that we were unable reduce the interacting region of the polymerases much further than our initial screen had revealed (Fig. 7), Additional file 5. We also noticed that different regions of Pol1A and Pol1B were able to interact with the small region of Twinkle we had identified. Spot dilutions of each interaction allude to a much stronger association between Pol1A and Twinkle versus Pol1B with Twinkle.

### Thermophoresis analysis of Twinkle/DNA polymerase interactions

Thermophoresis is a biochemical assay that measures interaction between molecules through the use of a temperature gradient. Molecules, in this case proteins, are suspended in liquid which is heated by a laser. The laser is focused to heat a specific point of the liquid, creating a temperature gradient in the surrounding area. Movement of molecules through this temperature gradient indicates the relative size of the molecules with smaller molecules moving quicker than larger ones. If two molecules or proteins interact with each other, the larger size of the complex retards its movement through the liquid. This movement can be used to calculate dissociation constants of two interacting molecules. Using thermophoresis we were able to determine the binding strength between Twinkle and different regions of Pol1B.

**Fig. 8:**
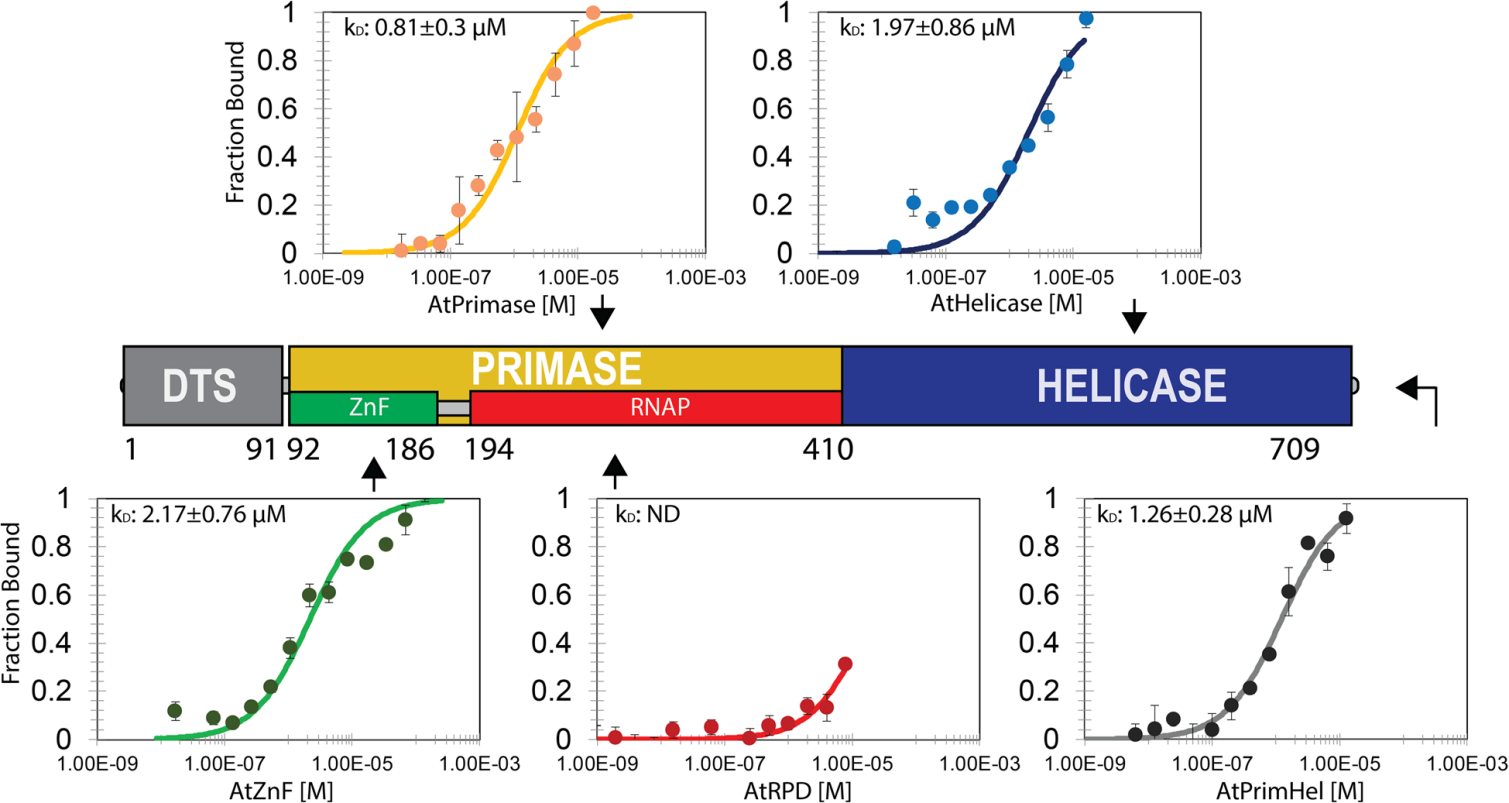
Thermophoresis binding of DNA Pol1B with Twinkle domains. The DNA polymerase 1B was labeled as the target protein at 20 nM concentration and assayed in microscale thermophoresis experiments with Twinkle, the primase and helicase domains, and the zinc finger subdomain. The thermophoretic data was fitted to the Kd equation described in materials and methods. The zinc finger subdomain was able to bind to the PolB and a Kd was measured at 2.17 μM. The RNAP subdomain (red) did not show binding (data not shown). The primase and helicase domain were able interact with the polymerase with a Kd of 0.81 and 1.97 μM respectively. The full-length protein interacts with a Kd of 1.26 μM. All proteins were titrated in 16 serial dilutions from different concentrations. Graphs were plotted at x axis with enzyme concentration and at the y axis with normalized fluorescence. Error bars represent the standard error for three measurements

Although the yeast-two hybrid experiments showed a stronger interaction between Pol1A and Twinkle, Pol1B was used to determine the binding constant with Twinkle and its different structural modules because this recombinant protein had a better yield during protein purification and because of the 70% amino acid identity present between Pol1A and Pol1B. In this experiment a polyhistidine-tagged Pol1B and the ligands (full-length Twinkle, Zn^++^ Finger domain, RNAP domain, primase module and helicase module) without a histidine tag were used. The his-tagged Pol1B was labeled with a fluorescent dye and the amount was kept constant at 10 nM during the binding experiments. After incubation with increasing concentrations of ligand, samples were loaded into MST standard capillaries. Pol1B showed specific binding to full length Twinkle, the primase and helicase domains, and the zinc finger subdomain. The RNAP region did not show appreciable interaction with Pol1B (data not shown; region indicated in Fig. 8). The fitted values from the thermophoretic analysis yielded dissociation constants for the ligand partners Pol1B-Twinkle of 1.26 μM, Pol1B-Primase of 0.81 μM, pol1B-Helicase of 1.97 μM, pol1B-zf of 2.17 μM (Fig. 8). These values are comparable with the binding affinities between T7 DNA polymerase and the bifunctional T7 primase-helicase [22–24]. The dissociation constants for this interaction are in the order of 0.44 μM in the presence of DNA [25]. The DNA primase and DNA helicase activities are encoded as two independent polypeptides in bacteria. In *Bacillus subtilis* the dnaG primase protein has been documented to interact with the DNA polymerase dnaE helicase with a dissociation constant of 0.75 μM [26]. Finally, zinc finger containing proteins have been described as interacting partners with DNA polymerases, in which cases the zinc finger is critical for physical interaction between both proteins [27, 28]. The confirmation of the interaction between the Twinkle and Pol1B provide further support for the role of these enzymes in replicating the *Arabidopsis thaliana* organellar genome.

### Twinkle T-DNA mutant analysis

**Fig. 9:**
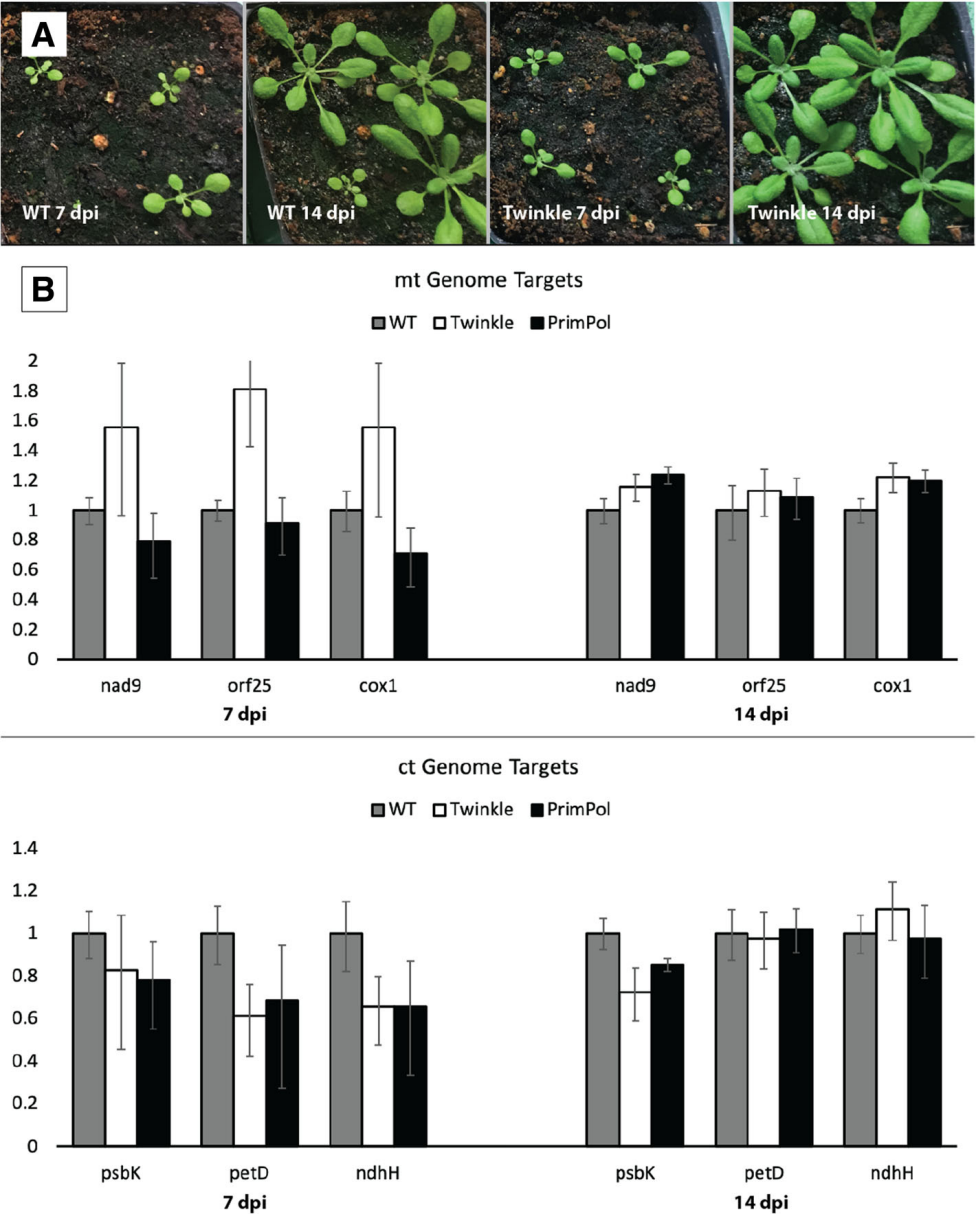
Growth and genome copy number differences between WT and Twinkle mutant Arabidopsis plants. (**a**) No distinguishable phenotype can be observed between WT and Twinkle mutant plants. (**b**) Relative qPCR analysis reveals no significant difference in mitochondrial and chloroplast genome copy numbers in Twinkle and PrimPol T-DNA mutants compared to wild-type plants. The y-axis represents a percentage of the genome copy numbers present in mitochondria and chloroplasts relative to WT plants. Error bars represent 95% confidence intervals

Having established positive interactions between Twinkle and the DNA polymerases, we obtained T-DNA mutants of the *Arabidopsis* Twinkle (T-DNA mutant SALK_152246) and another potential primase protein, PrimPol (At5g52800; T-DNA insertion line Salk_090163)*.* Surprisingly, the Twinkle mutants appear unaffected by the loss of this protein (Fig. 9a); PrimPol mutants also showed no noticeable phenotype (not shown). This was followed with relative qPCR analysis of genome copy numbers, which showed no significant change in mitochondrial or chloroplast DNA levels relative to the nuclear genome due to the Twinkle or PrimPol insertion mutations (Fig. 9b). Considering that Twinkle homologs are essential for both T7 phage and animal mitochondria genome maintenance, we were surprised to see no apparent phenotype or genome copy number changes in the mutant plants compared to wild-type plants.

## Discussion

We have identified a region of *Arabidopsis* Twinkle DNA primase/helicase that is crucial for interaction with the two organellar DNA polymerases. Within this region, mutation of several key residues results in complete dissociation from Pol1A or Pol1B as determined by yeast-two-hybrid analysis. We have also shown that mutation of key residues in Pol1A disrupts interaction with Twinkle. While the key residues in Twinkle are fairly closely clustered, these key residues in the DNA polymerases are spaced much further apart. We suspect this was the main reason we could not produce a smaller truncation of Pol1A or Pol1B that maintained interaction with Twinkle, whereas the interacting region of Twinkle was localized to a very narrow region of the protein. This supports our hypothesis that the N-terminal region of Twinkle coordinates an interaction with Pol1A or Pol1B. However, it does not confirm that only the N-terminal region of the DNA polymerases is crucial for positive interaction with Twinkle. Results from our previous screens also show this same region of Twinkle associates with SSB1, supporting the idea that Twinkle coordinates the assembly of a minimal DNA replisome. As most vascular plants possess the same orthologs of these organellar replication proteins, this pattern is likely to be repeated in higher plant chloroplasts and mitochondria of other species.

Although we have provided evidence showing that Twinkle and the DNA polymerases likely form a minimal DNA replisome, we do not know if this is the sole DNA replisome utilized in plant organelles. Additionally, other proteins may provide accessory functions such as in *E. coli* [29, 30]. For example, almost no research has been performed on primer removal. Two 5 ‘-3’ exonucleases have been identified in *Arabidopsis* organelles [31, 32], one localizing to the mitochondria and one localizing to the chloroplasts, but there not have been any studies on their mechanisms of action. Ribonucleases have been reported in plant mitochondria and chloroplasts, but their characterized functions are in mRNA and tRNA processing [33]. We have also shown strong interactions of *Arabidopsis* Twinkle with SSB1, which may form a crucial part of a DNA replisome.

Pol1A and Pol1B have been shown to be processive enough to replicate an entire organelle genome equivalent [17, 34]. This is much more processive than *E. coli* DNA polymerase I, which is involved mostly in Okazaki fragment processing and DNA repair and cannot replicate much more than several dozen bases [35]. By itself, T7 DNA polymerase gp5 also lacks processivity, but when bound to *E. coli* thioredoxin it becomes highly processive. Interestingly, recombinant versions of *E. coli* DNA polymerase I that are able to bind thioredoxin display a dramatic increase in processivity [36]. Plant organelle DNA polymerases may also bind thioredoxin or other processivity factor to achieve their greater processivity, but this has never been shown. If this occurs, it would help explain why these enzymes possess much greater processivity than *E. coli* DNA polymerase I, although they are otherwise quite similar in sequence and function.

To date, no other organellar DNA polymerases have been identified in plant chloroplasts and mitochondria other than Pol1A and Pol1B. In addition to DNA polymerase(s), determining what helps unwind and prime DNA for replication is crucial to our understanding of DNA replication in plant organelles. We have demonstrated an association between the DNA polymerases and Twinkle suggesting that these two proteins are part of the DNA replisome in these organelles. However, homozygous Twinkle T-DNA insertion mutants in *Arabidopsis* show no noticeable defect in plant growth and there is no noticeable decrease in organelle genome copy number compared to wild-type plants (Fig. 9). The plants grow similarly to WT. This is puzzling, as similar interactions between T7 gp4 helicase-primase are essential for processive replication of phage DNA [24]. In addition, conditional Twinkle knockout mice fail to survive and display a rapid depletion of mitochondrial DNA in both heart and skeletal muscle tissue [37]. If Twinkle knockout plants grow well, this strongly suggests that another protein may provide the primase activity required for DNA polymerases to function. However, previous work has shown that Twinkle efficiently primes DNA synthesis by Pol1A and Pol1B [12]. This same study demonstrated that *Arabidopsis* Twinkle cannot prime T7 or *E. coli* DNA polymerase I. We and others (Brieba and Morley, unpublished observations) have shown that plants can survive with almost no visible growth defects if they have at least one functional organellar DNA polymerase. Despite this lack of phenotype, mutating Pol1A or Pol1B leads to an approximate 30% decrease in organelle genome copy number [38]. Growth at these decreased organelle genome copy numbers does not appear to be affected by low light conditions or drought (data not shown).

A simple explanation for the normal growth of Twinkle knockout plants grow normally is that there is another DNA replication protein that is either 1) the main helicase/primase active at replication forks or 2) another protein compensates for the loss of Twinkle activity. In either case, there is an apparent redundancy in Arabidopsis for Twinkle function. Other candidates include Twinky (At1g30660), a truncated version of Twinkle that retains only the DNA primase domain, and PrimPol (At5g52800), a unique primase/polymerase protein. However, T-DNA insertion mutations in either of these proteins also lead to no discernable growth defect (unpublished observations). In any case, Pol1A and Pol1B must be involved, since they are the only DNA polymerases found in the organelles and we and others have been unable to make double homozygous mutants in both genes.

In previous studies, we reported that plants with Pol1B mutations had a greater decrease in organelle genome copy number relative to plants with Pol1A mutations, particularly in mitochondria [38, 39]. We also observed a slight delay in growth of these plants. This suggests that Pol1B may be more important for DNA replication in the organelles than Pol1A. If the minimal replisome consists of Twinkle and a DNA polymerase, we expected based on our previous results that Twinkle would show a strong association with Pol1B. However, our research shows a stronger association with Pol1A. This could be explained by different scenarios: a strong interaction with Twinkle may be detrimental to DNA replication, or the roles of Pol1A and Pol1B may be less redundant and more distinct from each other. Supportive of this, one study demonstrated that Pol1B is primarily involved in DNA repair [17]. Other studies showed that both DNA polymerases can perform translesion repair but Pol1B is more effective at strand displacement than Pol1A [19, 40]. This suggests that Pol1A may be more involved in DNA replication whereas Pol1B is more involved in DNA repair in both mitochondria and chloroplasts. This contrasts with findings in maize, where only one of the two organellar DNA polymerases was shown to be responsible for replication of the maize plastid genome, as mutation of this single gene essentially abolished chloroplast DNA replication [41]. This maize mutant had about a two-fold reduction in mitochondrial DNA, implying that the second DNA polymerase may function in mitochondria.

In addition, recombination dependent replication (RDR) could explain the lack of a phenotype in Twinkle mutants. Extensive use of RDR in plant organelles has been demonstrated as a means of maintenance and repair of mitochondrial and plastid DNA [42]. This is especially true in mitochondria where direct and inverted sequence repeats of ≥50 bp are present throughout the genome. Mutations in recombination proteins lead to genome instability and are often lethal. One prominent example is RecA, a homolog of the bacterial replication protein RecA. Arabidopsis possess three RecA homologs that localize to the organelles (RecA1 [At1g79050], RecA2 [At2g19490], and RecA3 [At3g10140]). Mutations to these homologs lead to delayed phenotypes, increased recombination, and in the case of RecA2 plant death beyond the seedling stage [43–45]. Other recombination proteins that localize to the organelles include single stranded DNA binding proteins Whirly [46, 47] and OSB [48], and a MutS homolog called Msh1 [49]. Mutations to any of these proteins lead to an increase in illegitimate recombination and adversely affect plant development.

## Conclusions

We have used three independent approaches to confirm positive interactions between Twinkle and the organellar DNA polymerases Pol1A and Pol1B. We have used a classic molecular biology technique (yeast-two-hybrid), bioinformatics (DCA), and biochemistry (thermophoresis) to define important regions and residues that are key to these interactions. This three pronged approach provides confirmation of the interactions, and can be applied to other protein-protein interaction studies.

Further work examining the complete DNA replisome of plant organelles will identify other proteins involved in mitochondrial DNA replication in *Arabidopsis*, including when Twinkle is mutated. Candidates for study include Twinky, PrimPol, RecA, Whirly, and MutS. The same approach we have used to demonstrate the interaction between Twinkle and the DNA polymerases may reveal specific regions in these candidate proteins that are crucial for DNA replisome assembly and function.

## Methods

### Direct coupling analysis

We calculated both direct and indirect interactions between amino acid residues in the DNA polymerase (PolIA, At1g50840 and PolIB, At3g20540) and Twinkle (At1g30680) genes through a direct coupling analysis of sequences from 90 plant species. First, we manually downloaded each DNA polymerase and Twinkle gene from the National Center for Biotechnology Information (NCBI) [50], ensuring that each plant species included complete gene annotations for both genes. After separating the downloaded genes into two FASTA files, one for each gene, we performed a multiple sequence alignment on each FASTA file separately. We used the following CLUSTAL OMEGA [51] command, where ${INPUT} is the FASTA file containing the unaligned genes and ${OUTPUT} is the FASTA file containing the aligned genes:

clustalo -i ${INPUT} > ${OUTPUT}

A comparison of different multiple sequence aligners by Pais, Ruy [52] shows that CLUSTAL OMEGA performs relatively well for full-length gene sequences, similar to sequences used in our analysis.

Following the individual alignments of each set of sequences, we joined the aligned sequences for each species into a single FASTA file by adding 20 asparagine residues to the end of the Polymerase II gene followed by concatenating the Twinkle sequence for each species. This artificial buffer ensured that interactions identified between the two genes were not affected by combining the genes (e.g., residue proximity), and facilitated our ability to quickly differentiate between the two genes. We used a MATLAB implementation of DCA [53] to identify direct information and mutual information in each pairwise residue comparison between the two genes. The output file contains four columns: the position of the first amino acid residue, the position of the compared amino acid residue, the amount of direct information between the two residues, and the amount of mutual information between the two residues. We used the following command to perform the direct coupling analysis, where ${INPUT} is the combined FASTA file and ${OUTPUT} contains the direct information and mutual information for each pairwise comparison:

matlab -nodisplay -nojvm -nosplash -r "dca ${INPUT} ${OUTPUT}"

Since the amino acid residues reported in the DCA analysis came from the combined multiple sequence alignments (i.e., the position of the first residue of Twinkle starts after the 20 asparagine residues that follow the last residue of Polymerase II), we determined which residues corresponded to the residues in the original unaligned *Arabidopsis thaliana* sequences and extracted only those pairwise comparisons from the DCA output file. We then renumbered the amino acid residues to be congruent with the original unaligned sequences (i.e., the first residue of Polymerase II was labeled position one in the first column of the output file and the first residue of Twinkle was labeled position one in the second column of the output file). Finally, using these labels, we created a heatmap of the calculated mutual information using the matplotlib and scipy.interpolate.griddata libraries in Python version 2.7. We used the heatmap to visually identify areas of higher mutual information between the two genes.

### Yeast-two-hybrid analysis

Yeast-two-hybrid analysis was performed using materials and protocols from Clontech. This included using the Matchmaker gold strain of yeast for small scale yeast transformations following the lithium acetate protocol outlined by Clontech. Truncation libraries of Pol1A, Pol1B, Twinkle, and SSB1 (At4g11060) were created by cloning all possible truncations into the pGADT7 (prey) and pGBK (bait) plasmids (primers shown in Additional file 1: Table S1 and Additional file 2: Table S2)*.* These truncations were cloned first into *E. coli* to produce high copies and quantities of plasmid DNA. Harvested plasmid truncation libraries were then cloned into MATa and MATα haploid yeast. Appropriate haploid yeast libraries were subsequently mated and plated on media selective for positive interactions. Interacting truncations were identified by extracting DNA from yeast colonies, PCR amplifying plasmid DNA, and sequencing the PCR products to identify the truncation borders. All constructs identified in positive interactions were tested in both bait and prey plasmids, and for autoactivation by transforming the target protein against an empty bait or prey plasmid to eliminate the possibility of false positive interactions. For mating experiments, Matchmaker gold yeast was used in conjunction with an Arabidopsis library of normalized cDNAs purchased from Clontech.

Tri-alanine substitution mutants were created to test the effects of mutating the residues highlighted by DCA analysis. These mutants were created by utilizing ‘around-the-horn’ PCR. In this approach we used pGAD or pGBK plasmid with Twinkle or Pol1A correctly inserted as template DNA for PCR. Using a forward primer and a reverse primer with 5′ ends that base pair with the plasmid back-to-back with each other, we extended the length of the plasmid using high fidelity DNA polymerase. The forward primer possessed a tri-alanine mismatch region flanked by homologous sequences of ~ 15 bp for Twinkle or Pol1A. The resulting PCR product was a plasmid sized blunt ended DNA molecule possessing the tri-alanine substitution we had designed. The blunt ends of this molecule were ligated together and transformed into *E. coli.* Colonies were picked, grown and harvested for plasmid DNA which was checked for correct insertion of the tri-alanine substitution via Sanger sequencing. Once verified, the plasmids were transformed into yeast and measured for interaction using selective media.

### Thermophoresis

#### Cloning and expression

Twinkle lacking the first 91 codons was used as template for the construction of the DNA primase and helicase domains, and the RNAP and zinc finger subdomains. The primase domain (residues 92–410), RNAP and zinc finger subdomains (residues 194–410 and 92–186, respectively) were cloned into pET-19b and purified as described [12, 19]. The helicase domain (401–709) was cloned into the pCri-1b vector [54] and purified as described before, changing Tris-HCl to potassium phosphate pH7.0 in the buffer composition. Tags were removed using PreScission protease for the pET constructs and TEV protease for the construct in pCri-1b.

#### Microscale thermophoresis

Pol1B was labeled using NanoTemper’s Monolith His-Tag Labeling Kit RED-tris-NTA at 100 nM concentration. The fluorescent labeled protein was used at a constant concentration of 20 nM. The ligands were titrated against labeled Pol1B in 16 serial dilutions from 12.8 μM for Twinkle, 30 μM for the helicase domain, 70 μM for the primase domain, 30 μM for the RNAP subdomain and 270 μM for the zinc finger subdomain. The reactions were incubated in PBS buffer + 0.05% Tween. The measurements were performed using a NanoTemper Monolith NT.115p instrument and the analysis was conducted at 10% LED power and 50% MST power with standard capillaries. The data from the thermophoretic change was fitted according to the equation:

$$ (concentration)=U+\frac{\left(B-U\right)\left(C+{C}_T+{K}_d-\sqrt{{\left(C+{C}_T+{K}_d\right)}^2-4\ast C\ast {C}_T}\right)}{2{C}_T} $$

where U is the response value of the unbound state, B is the response value of the bound state and CT is the final concentration of the fluorescent molecule.

### qPCR

Leaf tissue was harvested from wild-type and Twinkle and Primpol mutant plants at 7 and 14 dpi. High quality DNA suitable for qPCR was isolated from tissue samples via a cetyltrimethylammonium bromide (CTAB) protocol adapted from Minas et al. [55]. Relative qPCR analysis was used to measure organelle genome copy number by measuring 3 mitochondrial and 3 chloroplast DNA targets (Additional file 3: Table S3). Mitochondrial targets included nad9 (NADH dehydrogenase iron-sulfur protein 3), orf25 (open reading frame, encodes plant b subunit of mitochondrial ATP synthase based on structural similarity), and cox1 (Cytochrome c oxidase subunit 1). Chloroplast targets included psbK (photosystem II reaction center protein K precursor), petD (Cytochrome b6-f complex subunit 4) and ndhH (NAD(P)H-quinone oxidoreductase subunit H). These targets are unique to mitochondria and chloroplast DNA to avoid overlap with the nuclear genome. AtRpoTp (phage-like RNA polymerase, nuclear encoded, plastid localized) was used as a standard to measure copy levels. Relative genome copy numbers were analyzed using an Applied Biosystems StepOne Plus qPCR machine and PowerUp SYBR green reagents. Technical and biological replicates were compiled and analyzed using the ∆∆Ct method [56].

## Additional files

Additional file 1: **Table S1** Primers used to create gene truncations. (DOCX 18 kb)

Additional file 2: **Table S2.** Primers used to create 10 residue truncations of Twinkle (DOCX 14 kb)

Additional file 3: **Table S3.** Primers used for qPCR analysis (DOCX 14 kb)

Additional file 4: **Figure S1. (PNG 343 kb)**

Additional file 5: **Figure S2. (PNG 1265 kb)**

## Data Availability

The datasets used and/or analyzed during the current study are available from the corresponding author on reasonable request. Alignment files are deposited in Treebase, ID 24443; http://purl.org/phylo/treebase/phylows/study/TB2:S24443 GenBank accession numbers for the *Arabidopsis thaliana* genes utilized in this study: DNA PolIA, At1g50840, GenBank accession NM_103965. DNA PolIB, At3g20540, GenBank accession NM_001203013. Twinkle, At1g30680, GenBank accession NM_179404. SSB1, At4g11060, GenBank accession NM_117176. PrimPol, At5g52800, GenBank accession NM_001161303. Twinky, At1g30660, GenBank accession NM_102803.

## Abbreviations

DCA: Direct coupling analysis
mt: Mitochondrial
PolIA: DNA polymerase IA
PolIB: DNA polymerase IB
SSB: Single-stranded DNA binding protein
zf: Zinc finger
