# Supplementary material for: Arabidopsis thaliana organelles mimic the T7 phage DNA replisome with specific interactions between Twinkle protein and DNA polymerases Pol1A and Pol1B
Source: BMC Plant Biol. 2019 Jun 6;19:241. doi: 10.1186/s12870-019-1854-3 (PMC6554949; doi:10.1186/s12870-019-1854-3)
Supplement: Supplementary file 1 — Table S1 Primers used to create gene truncations. (DOCX 18 kb) [file 12870_2019_1854_MOESM1_ESM.docx]

| **Supplementary Table 1. Primers used to create gene truncations** | | | |
| --- | --- | --- | --- |
| Primer name | Sequence | Pos. | Features/Restriction cut site |
| [A1]5' (1–) | GATC CCCGGG T* ATGGCCATGGGGGTTTC | 1 | SmaI |
| [A2]5' (36–) | GATC CCCGGG T* CCACTCCCTTCCTTCCTC | 36 | Excludes predicted signal peptide. SmaI |
| [A3]5' (275–) | GATC CCCGGG T* GCGAAGGATACCGTGGC | 275 | 15 AA before 3'-5' exonuclease domain. SmaI |
| [A4]5' (290–) | GATC CCCGGG T* GTCCATTCCTGTGATACAGAGGT | 290 | 3'-5' exonuclease domain border. SmaI |
| [A5]5' (640–) | GATC CCCGGG T* TGGCCCTCTGTAGGTGG | 640 | Starts at DNA pola superfamily domain. SmaI |
| [A6]5' (715–) | GATC CCCGGG T* TGTCATGCTATTGCCTCATTATGTG | 715 | 15 AA before polymerase domain. SmaI |
| [A7]5' (730–) | GATC CCCGGG T* TTGATCTCAAATTTTATTCTTCCGTTACAGG | 730 | Polymerase domain border. SmaI |
| [A8]3' (–290) | GATC GGATCC TTA GACATGATTCCTAAACTGATTCACGAG | 290 | 3'-5' exonuclease border. Stop codon. BamHI |
| [A9]3' (–305) | GATC GGATCC TTA TTCTTCCTTAACCTCAATCCCG | 305 | 15 AA into 3'-5' exonuclease. Stop codon. BamHI |
| [A10]3' (–523) | GATC GGATCC TTA TTTTACAAGAAGTTCACCAAAGGGTC | 523 | 3'-5' exonuclease border. Stop codon. BamHI |
| [A11]3' (–538) | GATC GGATCC TTA CTCAGCAAGATACTCTCTATCTACAAGT | 538 | 15 AA after 3'-5' exonuclease. Stop codon. BamHI |
| [A12]3' (–654) | GATC GGATCC TTA TTTCCCAGCTAACTCTTTCAAAACATC | 654 | 15 AA into DNA pola superfamily. Stop codon. BamHI |
| [A13]3' (–730) | GATC GGATCC TTA CAAAGAGTCTATAGAGCAAACTTCACA | 730 | Polymerase domain border. Stop codon. BamHI |
| [A14]3' (–745) | GATC GGATCC TTA GCCTGATACATTACTTCCCTGTAAC | 745 | 15 AA into polymerase domain. Stop codon. BamHI |
| [A15]3' (–1050) | GATC GGATCC TTA CTATTTGGCAGCATACCAGTTTTGA | 1050 | Last AA of Pol1A. Stop codon. BamHI |
|  |  |  |  |
| [B1]5' (1–) | GATC GAATTC ATGGGGGTTTCTCTTCGTCA | 1 | First AA of Pol1B. EcoRI |
| [B2]5' (30–) | GATC GAATTC GTCCCTCGCCGTCGAAT | 30 | Excludes predicted signal peptide. EcoRI |
| [B3]5' (249–) | GATC GAATTC AATGTGTCTAGTGCAAAGGAAACC | 249 | 15 AA before 3'-5' exonuclease. EcoRI |
| [B4]5' (264–) | GATC GAATTC TATAGGAATCTTGTCCATGCTTGC | 264 | 3'-5' exonuclease domain border. EcoRI |
| [B5]5' (618–) | GATC GAATTC GGCTGGCCCTCTGTTAG | 618 | DNA pola superfamily border. EcoRI |
| [B6]5' (699–) | GATC GAATTC TGCCATGCTATTGCTGCA | 699 | 15 AA before polymerase domain. EcoRI |
| [B7]5' (714–) | GATC GAATTC TTAATATCAAATTTTATCCTTCCTTTACAGGGAA | 714 | Polymerase domain border. EcoRI |
| [B8]3' (–264) | GATC GGATCC TTA ATATTGATTCATGAGCAGAGCCAC | 264 | 3'-5' exonuclease border. Stop codon. BamHI |
| [B9]3' (–279) | GATC GGATCC TTA ATCAATCCTGGATACCTCTGTATCG | 279 | 15 AA into 3'-5' exonuclease. Stop codon. BamHI |
| [B10]3' (–501) | GATC GGATCC TTA TTTGGCAAGAAGTTCACCAAAAG | 501 | 3'-5' exonuclease domain border. Stop codon. BamHI |
| [B11]3' (–516) | GATC GGATCC TTA CTGCGCCAAATAATCCCTATCT | 516 | 15 AA after 3'-5' exonuclease. Stop codon. BamHI |
| [B12]3' (–633) | GATC GGATCC TTA TTTCCCAGCTAAGGCTTTCAAG | 633 | 15 AA into DNA pola superfamily. Stop codon. BamHI |
| [B13]3' (–714) | GATC GGATCC TTA TAAGGAATCAATGGAGCAAACTTCAC | 714 | Polymerase domain border. Stop codon. BamHI |
| [B14]3' (–729) | GATC GGATCC TTA TCCTGACACGTTACTTCCCT | 729 | 15 AA into polymerase domain. Stop codon. BamHI |
| [B15]3' (–1034) | GATC GGATCC TTA TTATTTGCCAGCATACCAGTTCTG | 1034 | Last AA of Pol1B. Stop codon. BamHI |
|  |  |  |  |
| [S1]5' (1–) | GATC GAATTC ATGAACTCACTCGCCATTAGAGT | 1 | First AA of SSB1. EcoRI |
| [S2]5' (55–) | GATC GAATTC CTTCAACCTCATGGAGTTGATCC | 55 | 15 AA before SSB1 protein family domain. EcoRI |
| [S3]5' (70–) | GATC GAATTC GGTGTTCATAGGGCGATTATTTGT | 70 | SSB1 family domain border. EcoRI |
| [S4]3' (–70) | GATC GGATCC TTA ACCGCGAAATCCCCAAC | 70 | SSB1 family domain border. Stop codon. BamHI |
| [S5]3' (–85) | GATC GGATCC TTA TAACGGTGCTTGCCCTAC | 85 | 15 AA into SSB1 family domain. Stop codon. BamHI |
| [S6]3' (–175) | GATC GGATCC TTA ACGACGAACGCAAATCTCAG | 175 | SSB1 family domain border. Stop codon. BamHI |
| [S7]3' (–201) | GATC GGATCC TTA CTAAATCAATCCTTCTTTTAGCTCATCAAAAG | 201 | Last AA of SSB1. Stop codon. BamHI |
|  |  |  |  |
| [T1]5' (1–) | GATC CATATG ATGCGATTTTTGCTTCGTTTACCA | 1 | First AA of Twinkle. NdeI |
| [T2]5' (40–) | GATC CATATG TACCCTTCTTCTCCTTCTTATTCTTCA | 40 | Excludes signal peptide. NdeI |
| [T3]5' (265–) | GATC CATATG AAGACACGGAGGATCTTATATGGT | 265 | 15 AA before Uniprot primase domain. NdeI |
| [T4] 5' (280–) | GATC CATATG TCTGAAGTCATTATAGTTGAAGGGGA | 280 | Uniprot primase domain border. NdeI |
| [T5] 5' (405–) | GATC CATATG GCTGAGCCATATCCTATACTAGGA | 405 | 15 AA before helicase domain. NdeI |
| [T6] 5' (420–) | GATC CATATG TTTGATGAAATTGATGCCTACTATGATAGAAC | 420 | Helicase domain border. NdeI |
| [T7] 3' (–280) | GATC GAATTC TTA AGATGTTTTTTCTATGTCATCAAGACCA | 280 | Uniprot Primase domain border. Stop codon. EcoRI |
| [T8] 3' (–295) | GATC GAATTC TTA TTCCATTGCAAGTTTATCTATCTCCC | 295 | 15 AA into primase domain. Stop codon. EcoRI |
| [T9] 3' (–420) | GATC GAATTC TTA AAAGAAATCTTTGAAGGAGAATAATCCTAGTATAGG | 420 | Helicase domain border. Stop codon. EcoRI |
| [T10] 3' (–435) | GATC GAATTC TTA ATACTCGTGCCCATGTGTTC | 435 | 15 AA into helicase domain. Stop codon. EcoRI |
| [T11] 3' (–709) | GATC GAATTC TTA TCAGTACCGCTTGGGTGA | 709 | Last AA of Twinkle. Stop codon. EcoRI |
| *Extra thymine nucleotide added to keep truncation in frame with cloning plasmids | | | |
