# Supplementary material for: Arabidopsis thaliana organelles mimic the T7 phage DNA replisome with specific interactions between Twinkle protein and DNA polymerases Pol1A and Pol1B
Source: BMC Plant Biol. 2019 Jun 6;19:241. doi: 10.1186/s12870-019-1854-3 (PMC6554949; doi:10.1186/s12870-019-1854-3)
Supplement: Supplementary file 2 — Table S2. Primers used to create 10 residue truncations of Twinkle (DOCX 14 kb) [file 12870_2019_1854_MOESM2_ESM.docx]

| **Suppplementary Table 2. Primers used to create 10 residue truncations of Twinkle** | |
| --- | --- |
| Primer Name | Sequence |
| *Forward primers* | |
| [T2.1] 5' (AA:50–) | GATC CATATG AGACAAGTATCTTCCGTTTCTAGAAGA |
| [T2.2] 5' (AA:60–) | GATC CATATG CGACCAGTTTTGGCCTC |
| [T2.3] 5' (AA:70–) | GATC CATATG AAAAACAGTCCTTATTACCAAAGGAC |
| [T2.4] 5' (AA:80–) | GATC CATATG GGTTTATCATCTTACAATTCAATCCCC |
| [T2.5] 5' (AA:90–) | GATC CATATG GTCCCAACTCCTGTTGATACTG |
| [T2.6] 5' (AA:100–) | GATC CATATG GCAGATAAGAGGGTTGTTCTATCT |
| [T2.7] 5' (AA:110–) | GATC CATATG GTGACTTTGAGGCGTAAATTGG |
| [T2.8] 5' (AA:120–) | GATC CATATG GGAGTTGATGCTGAAAACTGC |
| [T2.9] 5' (AA:130–) | GATC CATATG CAACATAGTGGCTTGATATGTCC |
| [T2.10] 5' (AA:140–) | GATC CATATG GAAGGTGGAAACTCTGGAGA |
| [T2.11] 5' (AA:150–) | GATC CATATG TCTCTTTTTATAGCCCCTGATGG |
| [T2.12] 5' (AA:160–) | GATC CATATG GCTACATGGAATTGCTTTAGGG |
| [T2.13] 5' (AA:170–) | GATC CATATG GGGTTAAAAGGTGGAGTTCG |
| [T2.14] 5' (AA:180–) | GATC CATATG GGGTTGGCATCTGCTGAT |
| [T2.15] 5' (AA:190–) | GATC CATATG GTTGAAAGAAAAATTACGGTGGAGG |
| [T2.16] 5' (AA:200–) | GATC CATATG GAGCTAGAACCTCTCTGTGAT |
| [T2.17] 5' (AA:210–) | GATC CATATG GATTATTTCGCTGCAAGAGCG |
| [T2.18] 5' (AA:220–) | GATC CATATG AAAACACTCGAGAGAAATCGGG |
| [T2.19] 5' (AA:230–) | GATC CATATG AAAAGAATAGGTGACGAGATTGTAATTG |
| [T2.20] 5' (AA:240–) | GATC CATATG TTTACTTATTGGCAAAGAGGGGAG |
| [T2.21] 5' (AA:250–) | GATC CATATG AGTTGCAAGTACCGGTCTC |
| [T2.22] 5' (AA:260–) | GATC CATATG TTCTTTCAGGAAAGGAAGACACG |
| [T2.23] 5' (AA:270–) | GATC CATATG TTATATGGTCTTGATGACATAGAAAAAACA |
| [T2.24] 5' (AA:280–) | GATC CATATG TCTGAAGTCATTATAGTTGAAGGGG |
| [T2.25] 5' (AA:290–) | GATC CATATG GATAAACTTGCAATGGAAGAAGCT |
|  |  |
| *Reverse primers* | |
| [T10.25] 3' (AA:–185) | GATC GAATTC TTA ATCAGCAGATGCCAACCC |
| [T10.24] 3' (AA:–195) | GATC GAATTC TTA CGTAATTTTTCTTTCAACCTTCTCTATAGG |
| [T10.23] 3' (AA:–205) | GATC GAATTC TTA ACAGAGAGGTTCTAGCTCTATACC |
| [T10.22] 3' (AA:–215) | GATC GAATTC TTA TCTTGCAGCGAAATAATCTTGAATC |
| [T10.21] 3' (AA:–225) | GATC GAATTC TTA ATTTCTCTCGAGTGTTTTCCGT |
| [T10.20] 3' (AA:–235) | GATC GAATTC TTA CTCGTCACCTATTCTTTTCTGC |
| [T10.19] 3' (AA:–245) | GATC GAATTC TTA TCTTTGCCAATAAGTAAACGCAAT |
| [T10.18] 3' (AA:–255) | GATC GAATTC TTA AGACCGGTACTTGCAACTC |
| [T10.17] 3' (AA:–265) | GATC GAATTC TTA CTTCCTTTCCTGAAAGAACATCTTAG |
| [T10.16] 3' (AA:–275) | GATC GAATTC TTA GTCATCAAGACCATATAAGATCCTCC |
| [T10.15] 3' (AA:–285) | GATC GAATTC TTA AACTATAATGACTTCAGATGTTTTTTCTATGT |
| [T10.14] 3' (AA:–295) | GATC GAATTC TTA TTCCATTGCAAGTTTATCTATCTCCC |
| [T10.13] 3' (AA:–305) | GATC GAATTC TTA AACGGATACACAATTGAGAAAACCA |
| [T10.12] 3' (AA:–315) | GATC GAATTC TTA CGAAGAAACCTTCGCTGGA |
| [T10.11] 3' (AA:–325) | GATC GAATTC TTA CGTGTCCTTGTCTTCCGAT |
| [T10.10] 3' (AA:–335) | GATC GAATTC TTA GTCATTGCAATTCCATAGAAATTTATACT |
| [T10.9] 3' (AA:–345) | GATC GAATTC TTA AATAACAATTCGAGACGCCTTTTTTAG |
| [T10.8] 3' (AA:–355) | GATC GAATTC TTA AGCTTGACCAGGTCCATC |
| [T10.7] 3' (AA:–365) | GATC GAATTC TTA ACCCAAACGCCGTGC |
| [T10.6] 3' (AA:–375) | GATC GAATTC TTA CGGCCACTTGACACGC |
| [T10.5] 3' (AA:–385) | GATC GAATTC TTA ATCTTTAAAATGTTCATCCTCACTTTTCT |
| [T10.4] 3' (AA:–395) | GATC GAATTC TTA AGGTCCCTTAGACATAAGAACC |
| [T10.3] 3' (AA:–40) | GATC GAATTC TTA AGCATCTAAAATAGCTTCCTTGAGT |
| [T10.2] 3' (AA:–415) | GATC GAATTC TTA GGAGAATAATCCTAGTATAGGATATGGC |
| [T10.1] 3' (AA:–425) | GATC GAATTC TTA GGCATCAATTTCATCAAAGAAATCTTTG |
