# Supplementary material for: Arabidopsis thaliana organelles mimic the T7 phage DNA replisome with specific interactions between Twinkle protein and DNA polymerases Pol1A and Pol1B
Source: BMC Plant Biol. 2019 Jun 6;19:241. doi: 10.1186/s12870-019-1854-3 (PMC6554949; doi:10.1186/s12870-019-1854-3)
Supplement: Supplementary file 3 — Table S3. Primers used for qPCR analysis (DOCX 14 kb) [file 12870_2019_1854_MOESM3_ESM.docx]

| **Supplementary Table 1. Primers used for qPCR analysis** | | | |
| --- | --- | --- | --- |
| Primer name | Sequence | Genome targeted | Gene target |
| 5’ AtRpoTp | CTGAATGCAGGTCGAAACTCGGG | Nuclear | RNA polymerase |
| 3’ AtRpoTp | GCTTGGAAGCCGTCTGCTAGAAC | Nuclear |  |
| 5’ nad9 | GTGGGAGCGAGAAGTTTGGGATATG | Mitochondrial | NADH dehydrogenase |
| 3’ nad9 | GGGTCATCTCAATGGGTTCAGAAACC | Mitochondrial |  |
| 5’ orf25 | TCAAAGTGACTCTCGACGGGAGC | Mitochondrial | B subunit of ATP synthase |
| 3’ orf25 | TGCCACAAATTCGCAAGCTGATCC | Mitochondrial |  |
| 5’ cox1 | GAAGTAGGTAGCGGCACTGGG | Mitochondrial | Cytochrome c oxidase subunit 1 |
| 3’ cox1 | ATTCCAGGTCCACGCATGTTGAAG | Mitochondrial |  |
| 5’ psbK | GTCGCCAAATTGCCAGAGGC | Plastid | PSII K protein |
| 3’ psbK | CGGCTTGCCAAACAAAGGCTAAGAG | Plastid |  |
| 5’ petD | TATTACGGGGAACCCGCATGG | Plastid | Cytochrome b6-f complex subunit 4 |
| 3’ petD | GCAAAAGGATCCGCAGGTTCACC | Plastid |  |
| 5’ ndhH | GACTTCCAGGGGGTCCCTATGAG | Plastid | NAD(P)H-quinone oxidoreductase |
| 3’ ndhH | CCCAACTCCCCTTTTGGAGCTTC | Plastid |  |
