# Supplementary figures and images for: Arabidopsis thaliana organelles mimic the T7 phage DNA replisome with specific interactions between Twinkle protein and DNA polymerases Pol1A and Pol1B
Source: BMC Plant Biol. 2019 Jun 6;19:241. doi: 10.1186/s12870-019-1854-3 (PMC6554949; doi:10.1186/s12870-019-1854-3)

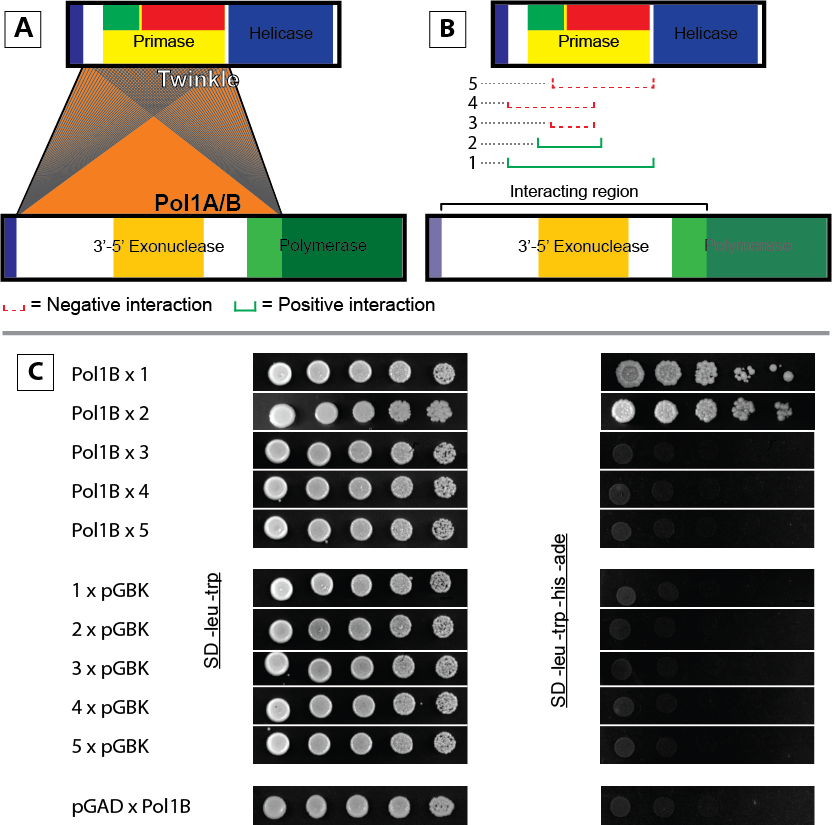

Supplement: Supplementary file 4 — Figure S1. (PNG 343 kb) [file 12870_2019_1854_MOESM4_ESM.png]

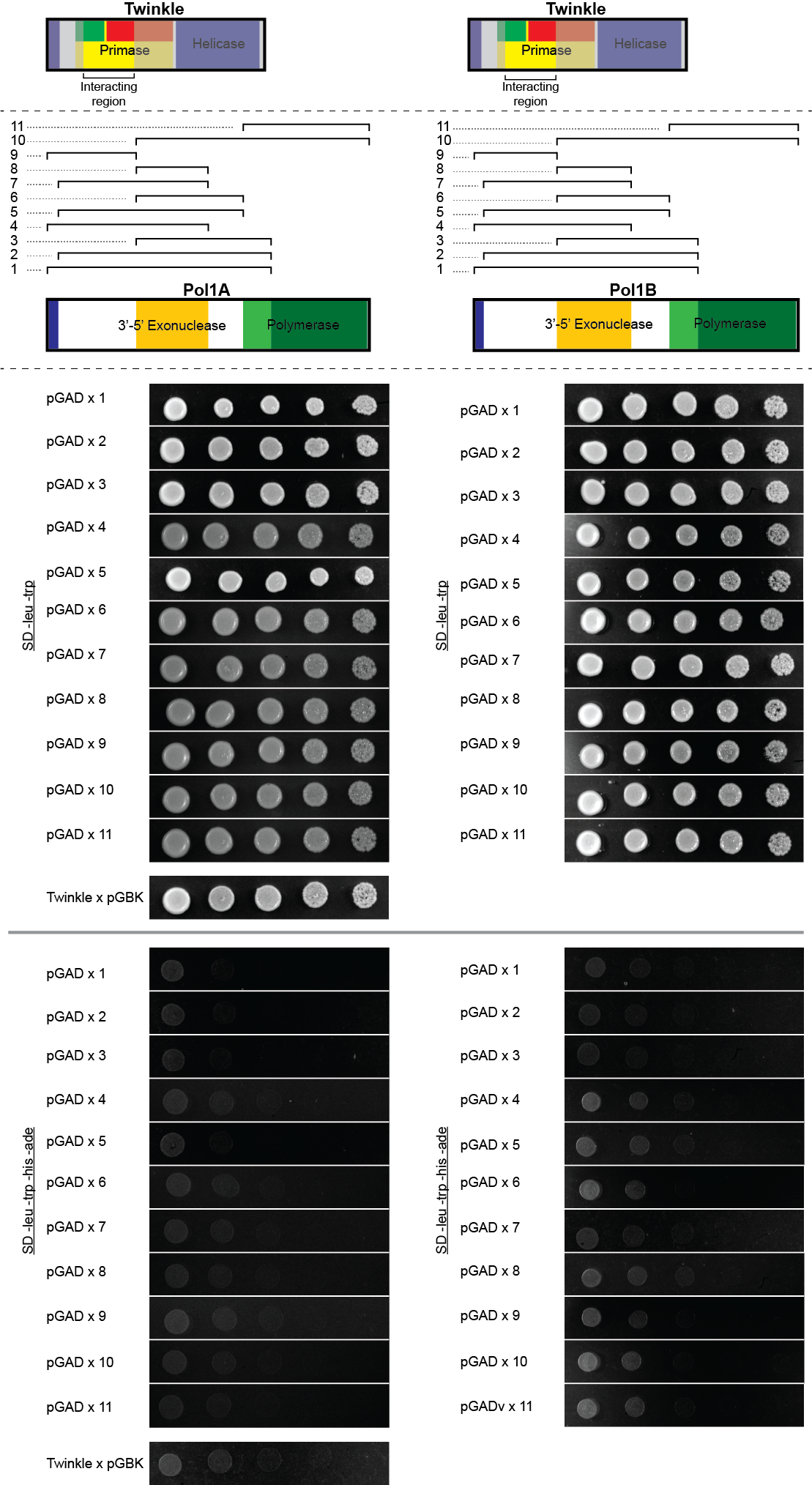

Supplement: Supplementary file 5 — Figure S2. (PNG 1265 kb) [file 12870_2019_1854_MOESM5_ESM.png]
